# Influence of resin cement shade on esthetic outcomes in ceramic veneers: a systematic review

**DOI:** 10.3389/fdmed.2026.1789416

**Published:** 2026-06-03

**Authors:** Subash Sharma, Aravind Kumar, Shruthi Rajagopal, Venkata Suresh Venkataiah

**Affiliations:** 1 Department of Aesthetics, Saveetha Dental College and Hospitals, Saveetha Institute of Medical and Technical Sciences, Saveetha University, Chennai, India; 2 Department of Orthodontics, Saveetha Dental College and Hospitals, Saveetha Institute of Medical and Technical Sciences, Saveetha University, Chennai, India; 3 Post Graduate Student Department of Conservative Dentistry and Endodontics, Saveetha Dental College and Hospitals, Saveetha Institute of Medical and Technical Sciences, Saveetha University, Chennai, India; 4 Clinical Sciences Department, Centre of Medical and Bio-Allied Health Science Research, College of Dentistry, Ajman University, Ajman, United Arab Emirates

**Keywords:** ceramic veneers, color stability, esthetic dentistry, final shade, *in vitro*, luting agents, optical properties, resin cements

## Abstract

**Introduction:**

The increasing demand for minimally invasive and highly esthetic dental restorations has led to the widespread use of ceramic laminate veneers, particularly ultrathin veneers. Due to their reduced thickness and high translucency, the final color of these restorations is influenced not only by the ceramic material but also by the underlying substrate and the resin cement used for luting. Understanding the optical contribution of resin cements is therefore essential for achieving predictable and long-term clinical success. This systematic review aimed to evaluate the influence of resin cement formulation and shade on the final color of ceramic laminate veneers.

**Methods:**

A comprehensive electronic search was conducted in the PubMed, Web of Science, ProQuest, and Google Scholar databases up to March 2023. *In vitro* studies assessing the effect of resin-based luting agents on the final color of ceramic laminate veneers using objective color measurement techniques, including the CIE L*a*b* color system and spectrophotometry, were included. Studies involving full-coverage restorations or subjective shade assessments were excluded. Risk of bias was assessed using the Quality Assessment Tool for *in vitro* Studies (QUIN), adapted for *in vitro* research.

**Results:**

Ten studies met the inclusion criteria. White opaque and high-chroma resin cements consistently resulted in greater color changes (ΔE > 3.3), particularly when used with thin or high-translucency ceramic veneers. In contrast, translucent and low-chroma resin cements produced more esthetically favorable outcomes. Veneer thickness emerged as a key modulating factor, with thinner veneers amplifying the influence of cement shade. Artificial aging studies demonstrated significant discoloration over time, highlighting concerns regarding long-term color stability. Risk-of-bias assessment indicated moderate methodological quality in most included studies.

**Discussion:**

The findings of this systematic review demonstrate that resin cement shade and translucency significantly affect the final color of ceramic laminate veneers. These effects are particularly pronounced in ultrathin and highly translucent restorations. Clinicians should therefore carefully consider resin cement selection when planning esthetic treatments, especially in the anterior region. However, the predominance of *in vitro* evidence underscores the need for well-designed clinical trials to validate these findings and establish standardized cementation protocols.

**Systematic Review Registration:**

PROSPERO CRD42023450540.

## Introduction

In contemporary restorative dentistry, ceramic laminate veneers have emerged as a minimally invasive and highly esthetic treatment option, offering excellent mimicry of natural dental tissues in terms of color, translucency, and light transmission (1, 2). Their success, however, relies not only on the ceramic material but also on the complex interplay of multiple optical factors—including the underlying tooth substrate and the luting agent (3, 4). Among these, the resin cement plays a pivotal role in determining the final shade of the restoration, particularly in cases involving thin, translucent veneers (5, 6).

Resin cements are designed to provide both mechanical retention and optical integration (7). These materials vary widely in composition, filler content, shade availability, and polymerization mode (light-cure, dual-cure, or self-cure), all of which can influence their optical behavior (7, 8). The final perceived shade of a cemented ceramic veneer is thus the result of a tri-layer optical interaction among the veneer, the luting agent, and the tooth substrate (5, 9). The resin cement's ability to transmit, reflect, or absorb light can dramatically alter the final esthetic appearance of the veneer—making cement selection a critical, yet often underappreciated, factor in restorative treatment planning (5, 9).

The challenge becomes more pronounced with the increasing demand for ultrathin veneers, where reduced ceramic thickness diminishes the material's ability to mask underlying color variations (3, 10). In such cases, the influence of the resin cement becomes magnified, potentially leading to perceptible or even clinically unacceptable color discrepancies (11, 12). While manufacturers provide a range of cement shades to accommodate different clinical scenarios, the color stability and predictability of these shades over time remain variable (6, 13).

Moreover, while try-in pastes are used to preview esthetic outcomes before definitive cementation, their reliability is questionable (14, 15). Curing-induced changes in refractive index and polymer matrix structure may result in a final shade that differs significantly from the pre-cure appearance, leading to patient dissatisfaction and restoration replacement (16).

Previous systematic reviews have evaluated the optical influence of resin cements on ceramic restorations; however, their scope has often been restricted to specific curing modes, limited ceramic systems, or earlier material generations, which may not fully reflect the diversity of contemporary luting agents and high-translucency ceramics. Perroni et al. assessed the influence of light-cured cements on veneer color, but variability in current shade systems, polymerization protocols, and ceramic materials warrants an updated synthesis of the available evidence (17).

To address these gaps, this systematic review synthesizes evidence from recent *in vitro* studies to primarily evaluate the influence of resin cement shade and translucency on the final color of ceramic laminate veneers, while secondarily considering formulation-related variables where these were consistently reported. Color change was assessed using standardized systems such as CIE L*a*b* and ΔE values. The aim is to provide clinicians with a clearer, evidence-based understanding of how resin cement selection affects esthetic outcomes and to highlight the need for standardized cementation protocols.

## Materials and methods

### Overview

This systematic review was conducted to evaluate the influence of resin cement formulation and shade on the final color of cemented ceramic laminate veneers. The methodology followed the PRISMA 2020 guidelines (18) and the Cochrane Handbook for Systematic Reviews of Interventions (19). The protocol was prospectively registered in the PROSPERO database (Registration ID: CRD42023450540). The research question addressed was: *Does the commercial formulation and shade of resin cement used for the luting of ceramic laminate veneers significantly affect the final shade of the cemented restoration?*

### Eligibility criteria

Eligibility criteria were framed using the PICOS model (Table 1). Studies were included if they were *in vitro* investigations evaluating the effect of resin-based luting agents on the final shade of ceramic laminate veneers using standardized quantitative techniques. Studies involving non-veneer restorations, those using qualitative assessment methods, or those lacking objective instrumentation for color analysis were excluded. Although one included study primarily evaluated fracture resistance, it also reported spectrophotometric color measurements that fulfilled the predefined outcome criteria and was therefore retained for qualitative synthesis.

**Table 1:** Selection criteria employed in the study.

Domain	Inclusion criteria	Exclusion criteria
Study design	*In vitro* experimental studies	Clinical studies, observational designs, or case reports
Intervention	Resin-based luting agents applied to ceramic laminate veneers	Cementation involving non-veneer ceramic restorations (e.g., crowns, inlays)
Outcome assessment	CIE L*a*b* color measurement using spectrophotometry or equivalent devices	Use of subjective or qualitative color-matching systems (e.g., Vita Shade Guide)
Analytical rigor	Reporting of statistical analysis of ΔE values	Absence of statistical comparison or reporting
Language	English-language publications	Non-English articles

### Data sources and search strategy

An electronic search was performed in PubMed, Web of Science, ProQuest, and Google Scholar databases through March 2023. No limitations were imposed on publication year. The search strategy incorporated MeSH terms and free-text terms related to ceramic laminate veneers and resin cement systems, including “ceramic veneers,” “resin cement,” “luting agent,” “CIE L*a*b*,” “spectrophotometry,” and “color difference.” Boolean logic and database-specific filters were employed to maximize relevance. Additional hand-searching of bibliographic references from relevant publications was also performed.

### Selection of studies

Two independent reviewers (SR and SS) conducted the screening process. Initial eligibility was determined by titles and abstracts, followed by full-text review of selected studies. Studies were included if they met all eligibility criteria. In instances of disagreement, consensus was reached through discussion, and unresolved decisions were referred to a third reviewer (TS). Where study data were incomplete or ambiguous, the corresponding authors were contacted for clarification.

### Data extraction

Data were extracted into a structured spreadsheet including: author and year, study design, sample characteristics, ceramic material type and thickness, commercial resin cement formulation and shade, method of polymerization, artificial aging protocol (if any), and the primary outcome (ΔE values indicating color change). Secondary variables, such as L*, a*, and b* value changes and significant intergroup differences, were also recorded where reported. Formulation-related variables (e.g., polymerization mode, resin matrix composition, and filler characteristics) were extracted where available and synthesized narratively because of inconsistent reporting and methodological heterogeneity among the included studies.

### Risk-of-Bias assessments

Risk of bias was assessed using the QUIN tool, which is specifically designed for *in vitro* dental research and has been developed to address sources of bias unique to laboratory-based experimental designs. This tool was selected in preference to instruments intended for clinical studies because it evaluates methodological domains relevant to standardization, reproducibility, and validity of outcome measurement in *in vitro* settings (20). This tool evaluates methodological rigor across eight domains: clarity of study aims, justification of sample size, use of a control group, standardization of procedures, blinding of the operator or examiner, appropriateness of statistical analysis, and the validity and reliability of outcome measures. The assessment was performed based on the completeness and transparency of reporting for each domain, and domain-level judgments were used to derive the overall risk-of-bias classification. Each study was evaluated based on the clarity of reporting within these domains, and an overall risk-of-bias judgment (low, moderate, or high) was assigned accordingly. The majority of studies demonstrated moderate methodological quality, with only three studies meeting criteria for low risk of bias. The most frequent limitations included lack of sample size justification and absence of examiner blinding. Overall risk of bias was categorized using a predefined approach based on the number of domains fulfilled: studies meeting ≥6 domains were considered low risk, those fulfilling 4–5 domains were classified as moderate risk, and those meeting ≤3 domains were categorized as high risk. The assessment was performed independently by two reviewers, and disagreements were resolved through discussion and, where necessary, consultation with a third reviewer to achieve consensus. A formal inter-reviewer agreement statistic (kappa) was not calculated.

### Meta-analytic feasibility

Due to substantial methodological heterogeneity among the included studies—specifically in resin cement systems, ceramic materials, sample preparation, luting techniques, and shade measurement protocols—a quantitative meta-analysis was not conducted. Instead, a qualitative synthesis was undertaken. The results were organized thematically to reflect the effects of material translucency, cement thickness, polymerization mode, and commercial formulation on color change outcomes. This heterogeneity also limited structured comparison of formulation-related variables across studies.

## Results

### Study selection

**Figure 1:**
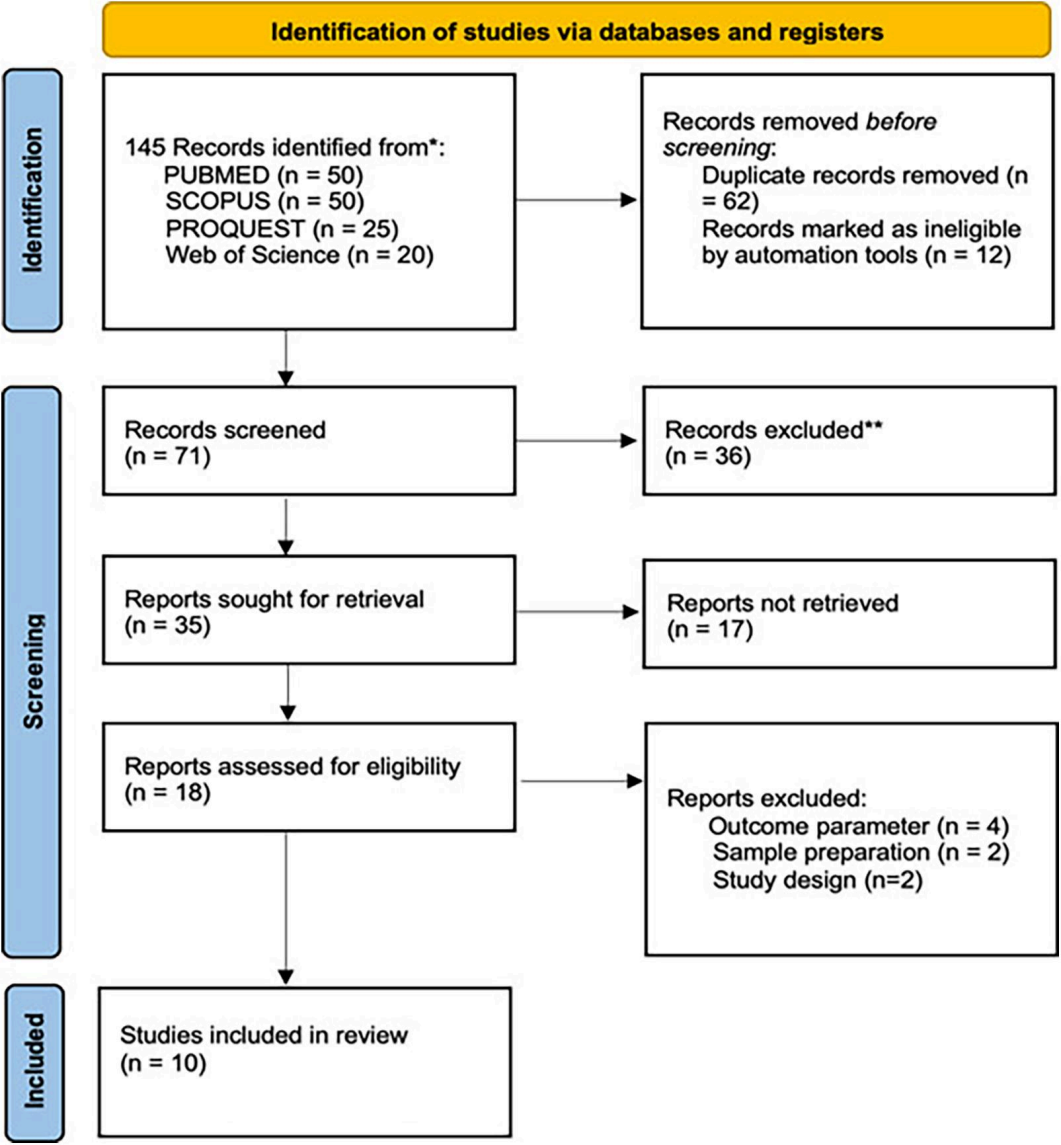
PRISMA flow diagram for the study.

The initial database search yielded 229 records. Following the removal of duplicates and non-English articles, 145 records remained. After screening titles and abstracts, 74 studies were excluded for not meeting the inclusion criteria. Subsequently, 71 full-text articles were assessed for eligibility. Of these, 61 were excluded due to reasons such as non-standardized color evaluation methods, inappropriate restoration types (e.g., crowns), and lack of statistical analysis. A total of 10 *in vitro* studies met all eligibility criteria and were included in the final qualitative synthesis. The study selection process is summarized in the PRISMA 2020 flow diagram (Figure 1).

### Characteristics of included studies

The included studies, published between 2011 and 2021, comprised diverse ceramic veneer systems and resin cement formulations, with sample sizes ranging from 10 to 392 specimens. All studies used objective colorimetric techniques, particularly CIE L*a*b* values, and reported color differences in ΔE values. Variations across studies included the type and thickness of ceramic material, resin cement formulation and shade, polymerization method (light-cure, dual-cure), and the application of artificial aging protocols. A detailed tabulation of study characteristics is presented in Table 2.

**Table 2:** Summary characteristics of included *in vitro* studies.

Author	Country	Sample size	Resin cement systems	Ceramic type	Color measurement method	Aging protocol
Alqahtani et al. (21)	Saudi Arabia	30	RelyX (TR, WO, A1, B0.5, A3)	Feldspathic	Spectrophotometer	None
Jankar et al. (26)	India	25	VarioLink II (Opaque, Translucent)	IPS Empress	Spectrophotometer	None
Lee et al. (24)	South Korea	168	VarioLink N (DB, DC) and Veneer (LP)	HT and LT e.max	Spectrophotometer	Thermocycling
Xing et al. (22)	China	72	RelyX (WO, A3, TR)	Likely e.max	Spectrophotometer	None
Hernandes et al. (5)	Brazil	10	VarioLink II (A1, A3)	HT and LT ceramics	Spectrophotometer	None
Hoorizad et al. (27)	Iran	50	Variolink and Choice 2	IPS e.max	Spectrophotometer	Artificial Aging
Turgut et al. (3)	Turkey	392	RelyX, Maxcem Elite, Variolink II	IPS Empress Esthetic	Spectrophotometer	None
Abdulelah Aldahlawi (23)	Saudi Arabia	108	VarioLink LC (Try-in and Final)	Veneers (1M1/2M2)	Spectrophotometer	None
Yildirim et al. (30)	Turkey	88	eCement (Transparent, Opaque)	ZLS, LDS	Spectrophotometer	Glycerin-simulated substrate
Chen et al. (4)	China	50	VarioLink, Panavia, RelyX	Ceramic disks (unspecified)	Spectrophotometer	None

### Risk-of-bias assessment

Among the ten included studies, five provided sample size justification, while seven established baseline comparability. Simulated intraoral conditions such as thermocycling or artificial aging were employed in five studies. Only one study explicitly addressed methodological error control. The majority of studies demonstrated moderate internal validity. A detailed summary of the risk-of-bias appraisal is presented in Table 3.

**Table 3:** QUIN-Based risk of bias assessment of included *in vitro* studies.

Author	Clearly stated aims	Sample size justified	Control group used	Standardization of procedures	Blinding of operator/examiner	Statistical analysis adequate	Outcome measures valid and reliable	Overall risk of bias
Alqahtani et al. (21)	Yes	No	Yes	Partial	No	Yes	Yes	Moderate
Jankar et al. (26)	Yes	No	Yes	Partial	No	Yes	Yes	Moderate
Lee et al. (24)	Yes	Yes	Yes	Yes	No	Yes	Yes	Low
Xing et al. (22)	Yes	Yes	Yes	Yes	No	Yes	Yes	Low
Hernandes et al. (5)	Yes	Yes	Yes	Partial	No	Yes	Yes	Moderate
Hoorizad et al. (27)	Yes	No	Yes	Yes	No	Yes	Yes	Moderate
Turgut et al. (3)	Yes	Yes	Yes	Yes	Yes	Yes	Yes	Low
Abdulelah Aldahlawi (23)	Yes	Yes	Yes	Yes	No	Yes	Yes	Low
Yildirim et al. (30)	Yes	No	Yes	Partial	No	Yes	Yes	Moderate
Chen et al. (4)	Yes	No	Yes	Partial	No	Yes	Yes	Moderate

**Table 4:** Summary of qualitative findings.

Author	Key variable studied	Key finding	ΔE range
Alqahtani et al. (21)	Resin cement shade (TR, WO, A1, B0.5, A3)	WO and A1 caused highest ΔE; translucent and A3 produced minimal changes	Up to ∼3.0
Jankar et al. (26)	Opaque vs. Translucent VarioLink cement	Opaque cement enhanced brightness; translucent increased yellowness	∼1.0–3.0
Lee et al. (24)	HT vs. LT ceramics with multiple resin shades and polymerization	Greatest color change with yellow-shaded dual-cure cement on HT ceramics	1.37–2.11
Xing et al. (22)	Veneer thickness and cement shade effects	0.5 mm veneers showed higher ΔE; WO and HV + 3 caused perceptible changes	<3.3 to >4.0
Hernandes et al. (5)	Resin cement shade under HT and LT ceramics	A3 shade under HT ceramic caused higher ΔE than A1	1.93–3.04
Hoorizad et al. (27)	Artificial aging on color stability of resin cement	Variolink cement showed highest ΔE after aging (ΔE = 10.4)	0.9–10.4
Turgut et al. (3)	Resin cement chroma and veneer thickness impact	Thin veneers and dark/opaque cements had most color change	1.6–3.4
Abdulelah Aldahlawi (23)	Try-in paste vs. final cement in varying thicknesses	Significant differences between try-in pastes and final cements after curing	0.32–11.49
Yildirim et al. (30)	Cement shade and ceramic type over tooth-colored backgrounds	Opaque cement caused larger ΔE on light backgrounds; material type had minor influence	2.0–6.0 (estimated)
Chen et al. (4)	Multiple resin cement shades and substrates	WO and HV + 3 led to greatest brightness and lowest chroma; exceeded clinical ΔE threshold	Up to 7.16

### Qualitative synthesis of findings

All ten studies consistently demonstrated that the final shade of cemented ceramic veneers was predominantly influenced by resin cement shade and translucency, while formulation-related factors were reported less consistently and could only be synthesized narratively. This synthesis is thematically structured to elucidate the influence of each variable on the final color outcome.

The shade and opacity of resin cement emerged as pivotal determinants of final esthetic results. Alqahtani et al. reported that white opaque cements resulted in the greatest color change (ΔE), followed by A1 and B0.5 shades, while translucent and A3 shades achieved greater blending and minimal perceptibility (21). This trend was corroborated by the findings of Chen and Xing et al., both of whom observed that white opaque and high-value cements (e.g., HV + 3, WO) significantly increased lightness (L*) and ΔE values, often surpassing the threshold of clinical acceptability (ΔE > 3.3) (4, 22). Turgut et al. similarly demonstrated that cements with high chroma, such as A3, as well as white opaque formulations, produced distinct visual shifts in shade, particularly when applied to 0.5 mm-thick ceramic veneers (3).

The effect of veneer thickness was also a consistent finding across the literature. Xing et al. and Abdulelah Aldahlawi both noted that thinner ceramic veneers (ranging from 0.3 mm to 0.5 mm) resulted in significantly greater color changes than thicker veneers of 1.0 mm or more (22, 23). Lee et al. and Hernandes et al. further demonstrated that high-translucency ceramics permitted greater light transmission and thereby accentuated the influence of underlying cement shade, particularly when yellow or opaque resin cements were employed (5, 24). In the study by Yildirim et al., the final color of the restoration was found to be predominantly governed by the resin cement when thin ceramics were used, regardless of the ceramic system (ZLS or LDS) (25).

In addition to thickness and translucency, the interaction between resin cement and underlying tooth structure or substrate was highlighted as a significant modulating factor. Jankar et al. found that cementing light-shade ceramic veneers with opaque resin cements enhanced the brightness and reduced the yellow chroma of the final restoration (26). Chen et al. and Yildirim et al. supported this observation by showing that opaque or high-value cement shades resulted in elevated ΔE values, particularly when placed over lighter background shades, indicating a strong cement-substrate interaction (4, 25).

The long-term color stability of cemented veneers was also examined in the context of artificial aging. Hoorizad et al. reported that resin cements such as Variolink exhibited the highest degree of discoloration following accelerated aging (ΔE = 10.4), while ceramic veneers alone exhibited minimal change (ΔE = 0.9), highlighting the impact of environmental stressors on resin cement esthetics (27). Abdulelah Aldahlawi drew attention to the considerable difference between the visual appearance of try-in pastes and the corresponding permanent cements after curing and aging, raising concerns about the reliability of pre-cementation shade evaluation (23).

In terms of clinical relevance, most studies adopted or referenced established perceptibility and acceptability thresholds, often defined as ΔE = 1.0 and ΔE = 3.3, as commonly reported in the dental color science literature (28). Studies such as those by Lee et al. and Turgut et al. indicated that translucent and low-chroma cements generally resulted in ΔE values below 3.3, suggesting acceptable clinical outcomes (3, 24). Conversely, high-opacity and high-chroma cements routinely exceeded these thresholds, indicating a greater risk of esthetic compromise.

Collectively, the findings across all ten studies converge on the conclusion that the optical properties of the resin cement, especially shade and opacity, play a determinative role in the final color of cemented ceramic veneers. This effect is amplified in restorations with high translucency and minimal thickness. Furthermore, the choice of luting agent should not be made in isolation but rather in consideration of the veneer's material properties, the substrate color, and the clinical requirement for long-term color stability. These results underscore the necessity for informed material selection and greater standardization in shade-matching protocols to achieve predictable esthetic outcomes in ceramic veneer restorations. The reported ΔE values represented either immediate post-cementation color differences or aging-induced discoloration depending on the study design, and these outcomes were interpreted separately in the present synthesis. Most included studies calculated color difference using the ΔE*ab formula, whereas some did not explicitly specify the method; none consistently reported the use of the ΔE00 system.

### Meta-analytic feasibility

A quantitative meta-analysis was not feasible due to substantial methodological and optical heterogeneity across the included studies. Veneer thickness ranged from approximately 0.3–1.0 mm, and multiple ceramic systems with different translucency levels were evaluated. Cement layer thickness and background substrates were not standardized, and artificial aging protocols varied in terms of thermocycling procedures, storage conditions, and duration. In addition, the color-difference calculation was inconsistently reported, with most studies using ΔE*ab, some not specifying the formula, and none uniformly applying ΔE00. Owing to these interstudy variations, statistical pooling was not considered appropriate, and the findings were synthesized qualitatively.

## Discussion

The results of this systematic review provide compelling evidence that the final shade of ceramic laminate veneers is substantially influenced by the shade, translucency, and optical behavior of the resin cement system used during luting. This finding reinforces a growing body of literature suggesting that the esthetic success of ceramic veneers is not solely dependent on the ceramic material, but also on the underlying adhesive layer, particularly when the restoration is thin or highly translucent.

These findings indicate that the resin cement layer should be regarded as an active optical component rather than a passive luting medium. The consistency of the observed trends across different ceramic systems and experimental protocols strengthens the material-science basis of the cement–ceramic–substrate interaction and supports a cement-integrated approach to shade selection, especially in ultrathin and high-translucency restorations.

The present review also places these observations within the broader optical framework of adhesive ceramic restorations, where light transmission, scattering, and absorption are governed by the refractive index compatibility between the ceramic, resin matrix, and filler particles. When these components are optically mismatched, interfacial scattering increases, resulting in perceptible color shifts. Conversely, closer refractive index matching promotes optical blending and improved esthetic integration.

The findings further underscore the clinical limitations of relying on try-in pastes to predict final outcomes. Aldahlawi documented marked ΔE differences between try-in pastes and cured resin cements, raising concern about the fidelity of visual shade evaluation in uncured states (23). These observations corroborate the concerns raised by Azer et al. and Douglas et al., who noted that polymerization alters refractive indices and optical interactions, especially in thicker or more opaque cement layers (29, 30). Formulation-related variables such as polymerization mode and filler content should be interpreted cautiously, as direct interstudy comparison was limited by inconsistent reporting.

The impact of artificial aging and environmental stressors on color stability was highlighted by Hoorizad et al., who reported significant discoloration following thermocycling (27). Similar conclusions with another study by Buchalla et al., who linked discoloration to hydrolytic degradation, incomplete polymerization, and breakdown of photoinitiators and fillers (31). These results suggest that clinicians must not only consider immediate esthetics but also long-term color stability when selecting resin cements.

In addition, the influence of aging should be interpreted in the context of resin chemistry and interfacial degradation. Water sorption, hydrolytic breakdown of the polymer matrix, and photoinitiator instability can progressively alter the optical properties of resin cements, thereby affecting long-term color stability even when the ceramic structure remains unchanged.

From a material science perspective, the optical behavior of resin cements is governed by multiple factors, including the refractive index, filler content, polymer matrix, and photoinitiator chemistry. Cements with a higher filler load and closer refractive index to ceramic demonstrate more favorable esthetic integration. The study by Pissaia et al. further emphasized the significance of polymerization mode and degree of conversion, with dual-cure cements exhibiting greater color shift post-curing than light-cured variants (6).

A notable strength of this review is its exclusive focus on *in vitro* studies employing objective, quantitative color measurement tools such as spectrophotometers based on the CIE L*a*b* system. These tools provide higher precision and eliminate observer bias inherent in traditional shade matching systems. The importance of enamel thickness reduction and objective instrumental color assessment has also been emphasized in previous restorative and shade-matching studies (32, 33). However, the absence of clinical trials limits generalizability, particularly in the context of oral fluids, thermal fluctuations, and patient-specific anatomical variability. In addition, incomplete reporting of ceramic material details in some of the included studies limited subgroup interpretation based on ceramic type.

The clinical implications of these findings are significant. Practitioners must consider the resin cement as an integral component of the esthetic outcome. In cases involving thin or highly translucent ceramic veneers, particularly in anterior regions, shade matching should incorporate the exact cement to be used—not merely a try-in paste—and cement selection must be tailored to the underlying tooth shade and veneer translucency. Standardization in veneer thickness and a deeper understanding of resin-ceramic optical interactions are critical for achieving predictable esthetic success.

Future research should address several gaps including long-term clinical trials evaluating color stability of different resin cements *in vivo*, development of predictive models incorporating veneer thickness, cement properties, and substrate color; and comparative studies evaluating newer bioinspired or nanofilled luting agents with improved optical stability. Additionally, standardized protocols for veneer preparation, cement handling, and light-curing would enhance reproducibility and evidence-based decision-making.

## Data Availability

The original contributions presented in the study are included in the article/Supplementary Material, further inquiries can be directed to the corresponding author.
